# Investigation of PALB2 Mutation and Correlation With Immunotherapy Biomarker in Chinese Non-Small Cell Lung Cancer Patients

**DOI:** 10.3389/fonc.2021.742833

**Published:** 2022-01-11

**Authors:** Jiexia Zhang, Shuangfeng Tang, Chunning Zhang, Mingyao Li, Yating Zheng, Xue Hu, Mengli Huang, Xiangyang Cheng

**Affiliations:** ^1^State Key Laboratory of Respiratory Disease, National Clinical Research Center for Respiratory Disease, Guangzhou Institute of Respiratory Health, the First Affiliated Hospital of Guangzhou Medical University, Guangzhou, China; ^2^Department of Oncology, MaoMing People’s Hospital, Maoming, China; ^3^Medical Department, 3D Medicines Inc., Shanghai, China

**Keywords:** *PALB2*, immunotherapy, HRR, DDR, NGS

## Abstract

**Background:**

*PALB2*, a gene in the homologous recombination repair (HRR) pathway of the DNA damage response (DDR), is associated with the efficacy of platinum-based chemotherapy, immunotherapy, and *PARP* inhibitor therapy in several tumors. However, the *PALB2* characteristics, its correlation with immunotherapy biomarker, and the prognostic effect of immunotherapy in non-small cell lung cancer (NSCLC) were unknown.

**Methods:**

Tumor tissue samples from advanced Chinese NSCLC patients were analyzed by next-generation sequencing (NGS) (panel on 381/733-gene). Tumor mutation burden (TMB) is defined as the total number of somatic non-synonymous mutations in the coding region. Microsatellite instability (MSI) was evaluated by NGS of 500 known MSI loci. Programmed Cell Death-Ligand 1 (PD-L1) expression was evaluated using immunohistochemistry (Dako 22C3 or SP263). One independent cohort (Rizvi2018.NSCLC.240.NGS cohort) containing genomic and clinical data from 240 patients with advanced NSCLC and two cohorts (the OAK and POPLAR study cohort) containing genomic and clinical data from 429 patients with advanced NSCLC were used to analyze the prognostic effect of *PALB2* on immunotherapy.

**Results:**

Genetic mutation of 5,227 NSCLC patients were analyzed using NGS, of which 162 (3.1%) harbored germline *PALB2* mutation (*PALB2^gmut^*) and 87 (1.66%) harbored somatic *PALB2* mutation (*PALB2*^smut^). In NSCLC patients with *PALB2^gmut^* and *PALB2^smut^*, the most frequently mutated gene was *TP53* (65%, 64%). *PALB2^smut^* (14.52 Muts/Mb) was associated with higher TMB (*p* < 0.001) than *PALB* wild-type (*PALB2^wt^*) (6.15 Muts/Mb). However, there was no significant difference in TMB between *PALB2^gmut^* (6.45 Muts/Mb) and *PALB2*^wt^ (6.15 Muts/Mb) (*p* = 0.64). There was no difference in PD-L1 expression among *PALB2^gmut^*, *PALB2^smut^*, and *PALB2^wt^*. In the Rizvi2018.NSCLC.240.NGS cohort, there was no difference in progression-free survival (PFS) (HR =1.06, *p* = 0.93) between *PALB2* mutation (3.15 months) and *PALB2^wt^* (3.17 months). The OAK and POPLAR study cohort of NSCLC patients showed that there was no difference in overall survival (OS) (HR =1.1, *p* = 0.75) between *PALB2* mutation (10.38 months) and *PALB2^wt^* (11.07 months).

**Conclusions:**

These findings suggest that *PALB2* may not be used as a biomarker for determining prognosis on immunotherapy in NSCLC.

## Introduction

The DNA damage response (DDR) is a collective term for the plethora of different intra- and intercellular signaling events and enzyme activities that result from the induction and detection of DNA damage (1). As a hot direction, there are many pieces of research related to DDR at present, which shows that DDR not only can predict the risk of breast cancer, ovarian cancer, and other cancers but also is related to the efficacy of various treatments, such as the presence of BRCA [a member of the homologous recombination repair (HRR) pathway] mutation that has been reported to correlate with the risk of breast cancer and the efficacy of PARP inhibitors, and the reports that multiple DDR pathway genes, including BRCA, predict the efficacy of immunotherapy for advanced urothelial carcinoma (2–4). According to previous literature, the DDR system comprises eight pathways, namely, mismatch repair (MMR), base excision repair (BER), checkpoint factors, Fanconi anemia (FA), HRR, nucleotide excision repair (NER), non-homologous end-joining (NEJ), and DNA translesion synthesis (TLS) (5). The current results show that the role of each pathway is different. For example, mutations in MLH1, MSH2, MSH6, or PMS2 in the MMR pathway can predict the immunotherapy benefit of patients with colorectal cancer (6), and PRO found and the TBCRC 048 study demonstrated, respectively, that Olaparib (PARP inhibitor) has a favorable benefit in prostate and breast cancer patients with HRR gene mutations (7, 8). *PALB2*, an important member of the HRR pathway, is frequently observed in cancer. In lung cancer, *PALB2* mutations occurred at 1.8% of cases, which is the highest rate among all cancer types (9). *PALB2* has been explored in the field of chemotherapy, and the presence of *PALB2* mutation has been reported to be correlated with improved clinical outcomes in non-small cell lung cancer (NSCLC) treated with platinum-based chemotherapy (10). None of the previously reported studies of the correlation between the DDR gene and lung cancer immunotherapy has independently verified *PALB2* (11–13). Therefore, it is necessary to analyze the *PALB2* mutation characteristics in the Chinese NSCLC population and demonstrate whether *PALB2* mutation is associated with immunotherapy responses. In this study, we attempted to analyze the characteristics and correlation with the immunotherapy biomarker of *PALB2* mutation among advanced Chinese NSCLC patients. Furthermore, the relationship between *PALB2* and response to immunotherapy was analyzed in a public cohort.

## Materials and Methods

### Clinical Cancer Specimens

A total of 5,227 advanced Chinese NSCLC patients from two centers (the First Affiliated Hospital of Guangzhou Medical University and MaoMing People`s Hospital) between January 2017 and January 2021 were included in the analysis. Formalin-fixed paraffin-embedded (FFPE) tumor specimens of NSCLC patients were used for next-generation sequencing (NGS) testing. The specimens were confirmed by hematoxylin and eosin (H&E) staining for a pathological diagnosis and were considered as qualified with a size ≥1 mm^3^ and the percentage of cancer cells should be over 20%. All procedures performed in this study involving human were in accordance with the Declaration of Helsinki (as revised in 2013). The study was approved by the Research Ethics Committee of the First Affiliated Hospital of Guangzhou University (Ethics Code: 2020-072).

### Next-Generation Sequencing

NGS was applied to guide subsequent treatment strategies. A 381/733-cancer gene panel was utilized for NGS as previously described (14) on Illumina Nextseq 500 to >500× coverage in 3DMed Clinical Laboratory Inc., a College of American Pathologists (CAP) and Clinical Laboratory Improvement Amendments (CLIA) approved laboratory of 3D Medicines Inc. Somatic and germline alterations were identified and clinical information were collected. Germline variants were identified by comparing each tumor tissue with the matched blood control. Pathogenic and very likely pathogenic mutations were interpreted by the bioinformatics specialist upon a joint consensus of the previous reports and the recommendation of the American College of Medical Genetics and Genomics and the Association for Molecular Pathology (ACMP-AMP) (15). PALB2 mutations were defined as the germline or somatic single-nucleotide variants (SNVs), copy number variations (CNVs), and fusion. SNVs include missense, nonframeshift, frameshift, splice, nonsense, and nonstop mutations; CNVs include gain and loss mutations.

TMB was defined as the number of nonsynonymous somatic SNVs and indels per megabase in examined coding regions, with driver mutations excluded. All SNVs and indels in the coding region of targeted genes, including missense, silent, stop gain, stop loss, in-frame, and frameshift mutations, were considered. High tumor mutational burden (TMB-H) was defined as greater than the median value.

One hundred microsatellite loci were selected for MSI determination and each assay, and the top 30 loci with the best coverage were included for the final MSI score calculation. An in-house developed R script was employed to evaluate the distribution of reading counts among various repeat lengths for each microsatellite locus of each sample. Any sample with an MSI score of ≥0.4 was classified as MSI-H, and MSS otherwise.

### PD-L1 Testing

FFPE tissue sections were subjected to assessment of PD-L1 expression using the PD-L1 immunohistochemistry (IHC) 22C3 pharmDx assay (Agilent Technologies) or PD-L1 IHC SP263 (Roche Diagnostics GmbH).

Staining for 22C3 was performed on the Dako Link-48 autostainer system at Teddy Clinical Research lab while staining for SP263 was performed on the Roche BenchMark Ultra platform at QIAGEN Suzhou Clinical Lab. PD-L1 expression was determined using Tumor Proportion Score (TPS), the proportion of viable tumor cells showing partial or complete membrane PD-L1 staining at any intensity. TPS ≥ 1% was considered PD-L1 positive.

### Immune Cohort Analysis

Genomic and clinic data of public cohorts involving NSCLC patients receiving immunotherapy (OAK study cohort; POPLAR study cohort; Rizvi2018.NSCLC.240.NGS cohort) were analyzed. OS/PFS were analyzed in R-3.6.0 using the Survival package. Meta-analysis was performed in R-3.6.0 using the Meta package.

### Statistical Analysis

For normally distributed continuous variables, Student’s *t*-test was used to determine the differences between the two groups; otherwise, use the Mann–Whitney *U* test. Fisher’s exact test or the Chi-square test was used to identify the association of two categorical variables. All reported *p*-values were two-tailed, and *p* < 0.05 was considered significant unless otherwise specified. All analyses and graphs in the present study were performed by R 3.6.0.

## Results

### Patients’ Characteristics and Prevalent *PALB2* Mutations Across NSCLC

A total of 5,227 patients with NSCLC from two centers (the First Affiliated Hospital of Guangzhou Medical University and MaoMing People`s Hospital) were analyzed using NGS; the baseline characteristics of the patients are shown in **Table 1**; 3.1% (162/5227) harbored germline *PALB2* mutation (*PALB2^gmut^*) and 1.66% (87/5227) harbored somatic *PALB2* mutation (*PALB2^smut^*).

**Table 1:** All patient demographics and baseline characteristics.

Characteristics	*PALB2^gmut^*(162)	*PALB2^smut^*(87)
Age, median (IQR range)	62.5 (25–83)	61 (36–86)
Sex		
Female	105 (64.8%)	73 (83.9%)
Male	57 (35.2%)	14 (16.1%)
MSI status		
MSI-H	0 (0.0%)	1 (1.2%)
MSS/MSI-L	162 (100.0%)	84 (98.8%)
N/A	0	2
TMB, median (IQR range)	6.5 (0–60.4839)	14.5 (1.67598–75.8064)
PD-L1 (%)		
<1	66 (50.4%)	30 (50.0%)
≥1	67 (49.6%)	30 (50.0%)
N/A	29	27
Pathology		
LUAD	111 (68.5%)	47 (54.0%)
LUSC	26 (16.0%)	22 (25.0%)
LUAS	1 (0.6%)	2 (2.3%)
Others	24 (14.8%)	16 (18.4%)

N/A indicate that the patient has not been tested or that the test result is unqualified. Pathology abbreviations: LUAD, lung adenocarcinoma; LUSC, lung squamous cell carcinoma; LUAS, lung adenosquamous carcinoma.

**Figure 1:**
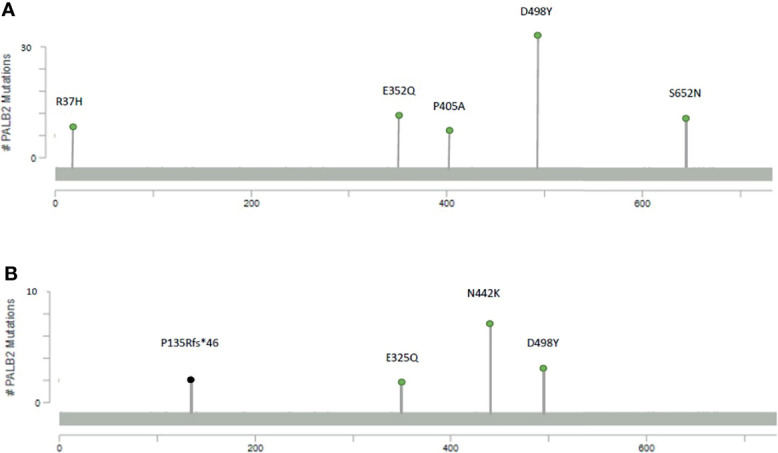
Mutational maps of the *PALB2* with the most frequent mutations, including **(A)** germline mutations and **(B)** somatic mutations.

In the *PALB2^gmut^* group, the most common variant is the missense mutation D498Y with 32 recurrences, followed by missense mutation S652N with 10 recurrences and missense mutation E352Q with 9 recurrences (**Figure 1A**). In the *PALB2^smut^* group, the most common variant is the missense mutation of N442K with 7 recurrences, followed by copy number loss and missense mutation of D498Y, each of which had 3 recurrences (**Figure 1B**).

**Figure 2:**
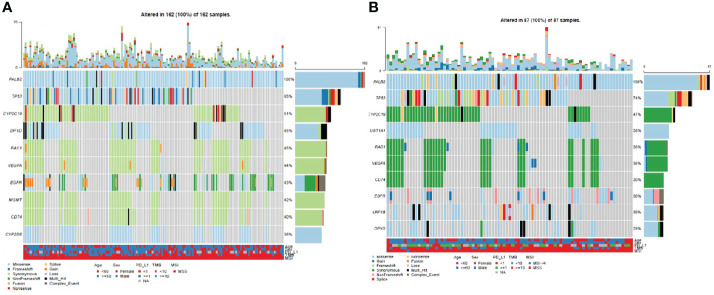
Waterfall plot (oncoplot) of variants in NSCLC patients with *PALB2* mutations, including **(A)** germline mutations and **(B)** somatic mutations.

Through statistical analysis of all variations, in NSCLC patients with *PALB2^gmut^*, the most frequently mutated gene was *TP53* (65%), followed by *CYP2C19* (51%), *DPYD* (45%), *RAC1* (45%), *VEGFA* (44%), *EGFR* (43%), *MGMT* (42%), and *CD74* (40%) (**Figure 2A**); the mutation frequency of *TP53* was the highest in NSCLC with *PALB2^smut^* (64%), followed by *CYP2C19* (47%), *UGT1A1* (38%), *RAC1* (36%), *VEGFA* (36%), *CD74* (36%), *EGFR* (30%), and *LRP1B* (30%) (**Figure 2B**). Detailed variation information can be found in the Supplementary Information (**Supplementary Table 1**).

### The Association of *PALB2* Mutation and TMB, MSI-H, and PD-L1

**Figure 3:**
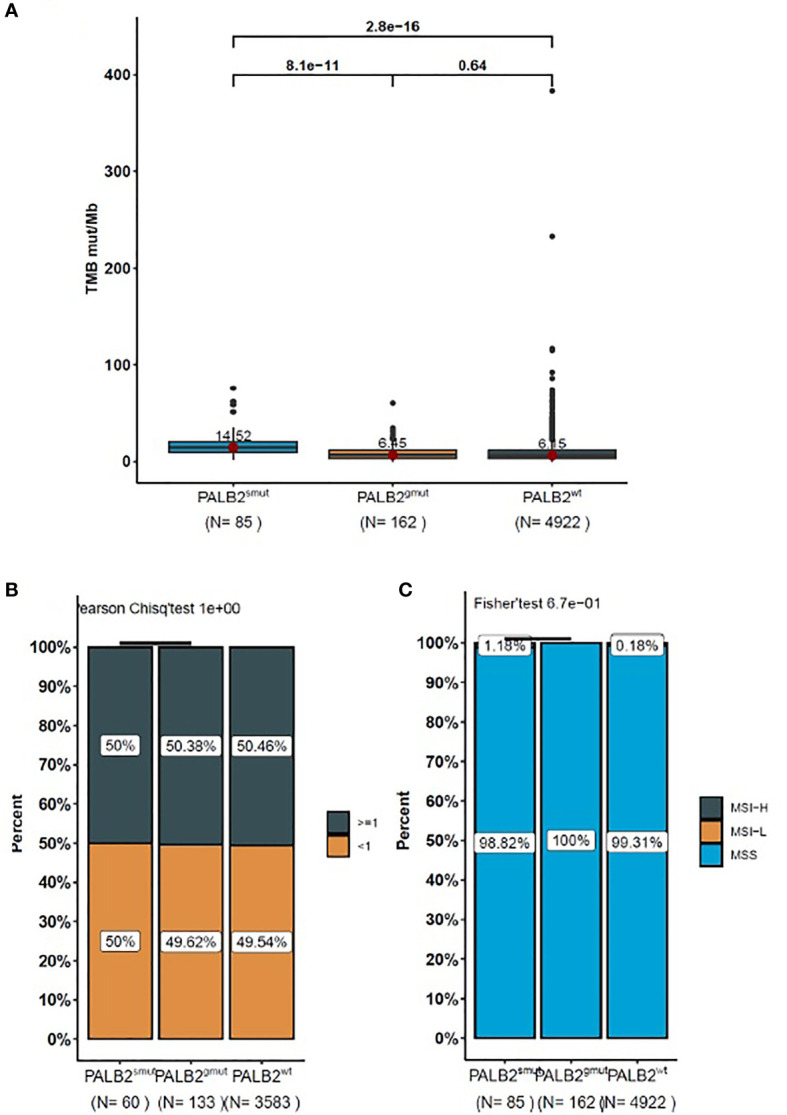
The box-plot for the level of **(A)** TMB, **(B)** PD-L1, and **(C)** MSI in NSCLC patients with *PALB2* mutations. The data presented in panels **(A–C)** represent the mean values of four samples (± standard deviation) and were analyzed with Student’s *t*-test.

*PALB2^smut^* (14.52 Muts/Mb) was associated with higher TMB (*p* < 0.001) than PALB wild type (*PALB2^wt^*) (6.15 Muts/Mb). However, there was no significant difference in TMB between *PALB2^gmut^* (6.45 Muts/Mb) and *PALB2^wt^* (6.15 Muts/Mb) (*p* = 0.64) (**Figure 3A**). There was no difference in PD-L1 expression among *PALB2^gmut^*, *PALB2^smut^*, and *PALB2^wt^*, which did not find any correlation with PD-L1 expression (**Figure 3B**). Similar to PD-L1, the variation in *PALB2* was not associated with MSI (**Figure 3C**).

### The Association of *PALB2* Mutation and Immunotherapy

We also analyzed the association between *PALB2* mutations and patient prognosis after immunotherapy. In the Rizvi2018.NSCLC.240.NGS cohort, there was no difference in progression-free survival (PFS) (HR =1.06, *p* = 0.93) between *PALB2* mutation (3.15 months) and *PALB2^wt^* (3.17 months). The OAK and POPLAR study cohort of NSCLC patients showed that there was no difference in overall survival (OS) (HR = 1.1, *p* = 0.75) between *PALB2* mutation (10.38 months) and *PALB2^wt^* (11.07 months).

## Discussion

The results showed that neither PALB2^smut^ nor PALB2^gmut^ was associated with these immunotherapy biomarkers, except that PALB2^smut^ was associated with significantly higher TMB. PALB2 mutations are not associated with the prognosis of immunotherapy in NSCLC patients.

Since the 2017 V3 version of the NCCN breast cancer guidelines for the first time added the PARP inhibitor olaparib as a treatment option for patients with HER-2 negative BRCA1/2 (members of the HRR pathway) mutations (16), the significance of the DDR gene (including the HRR gene) in the guidance of therapy has attracted more and more attention, including multiple directions of chemotherapy, targeted therapy, and immunotherapy. Similarly, the prognostic impact of HRR gene on NSCLC treatment has also been confirmed; for example, a study published in *Cancer Res* in 2018 found a correlation between DDR pathway genes and immunotherapy efficacy, but mostly focus on co-mutations from two kinds of mutations of DDR pathway, and do not discuss individual genes (11). Similarly, a study of 266 NSCLC patients receiving immunotherapy published in *Clin Cancer Res* in 2020 showed that patients with mutations in any of DDR genes had significantly better prognosis than those without DDR mutations. Notably, the genes of the HRR pathway were also included in this study, but there are only two patients that harbored PALB2 (member of the HRR pathway) gene mutation, so the impact of PALB2 mutation on the prognosis of NSCLC patients was not analyzed. As the number of patients carrying each gene variation in the above study varied greatly and each subset was not analyzed, the level of evidence obtained was not high, which was also mentioned in the *Discussion* section (12). Therefore, this is not inconsistent with the conclusion in this study that PALB2 mutation is not associated with immunotherapy prognosis. In fact, the results of the recently published Imagyn050 study showed that ovarian cancer patients with BRCA1/2 gene mutations were insensitive to immunotherapy, suggesting that not all DDR or HRR genes can be considered prognostic factors for immunotherapy (17). Moreover, for the relationship between PALB^smut^ and TMB, PALB^smut^ may be an epiphenomenon of a high TMB, rather than causing themselves. So, the relationship between DDR or HRR genes including *PALB2* and immunotherapy cannot be generalized, and each gene needs more specific studies to prove its role. In addition, whether *PALB2* can produce synergistic effects with other gene variants in DDR is not explained in this study, and more studies are needed for further exploration.

In conclusion, *PALB2* mutation in this study was not associated with immunotherapy. These findings suggest that *PALB2* may not be a prognostic biomarker for NSCLC patients receiving immunotherapy. In our study, we only conducted statistical analysis on NSCLC patients with *PALB2*, so it has certain limitations and needs more studies to verify it.

## Data Availability Statement

The datasets presented in this article are not readily available because making data publicly available would compromise patient confidentiality, and sequencing data contain sequencing algorithm and other core trade information of 3D Medicines Inc. Requests to access the datasets should be directed to the corresponding author JZ (E-mail address: drzjxcn@126.com).

## Ethics Statement

The studies involving human participants were reviewed and approved by the Research Ethics Committee of the First Affiliated Hospital of Guangzhou University. Written informed consent for participation was not required for this study in accordance with the national legislation and the institutional requirements.

## Author Contributions

Conception and design: JZ, XC, and ST. Acquisition of data: ML, CZ, and MH. Analysis and interpretation of data: CZ and YZ. Writing, review, and/or revision of the manuscript: XH and YZ. Study supervision: JZ, XC, and ST. All authors contributed to the article and approved the submitted version.

## Funding

This study was supported by the Clinical Application and Translational Medicine Project of First Affiliated Hospital of Guangzhou Medical University (201515-gyfyy to JZ), the Beijing Medical and Health Foundation (B19116 to JZ), and Livzon Pharmaceutical Group Inc. to JZ.

## Conflict of Interest

YZ, XH, and MH are employed by the company 3D Medicines Inc.

The remaining authors declare that the research was conducted in the absence of any commercial or financial relationships that could be construed as a potential conflict of interest.

## Publisher’s Note

All claims expressed in this article are solely those of the authors and do not necessarily represent those of their affiliated organizations, or those of the publisher, the editors and the reviewers. Any product that may be evaluated in this article, or claim that may be made by its manufacturer, is not guaranteed or endorsed by the publisher.

## Abbreviations

NSCLC, Non-small cell lung cancer; DDR, DNA damage response ; HRR, Homologous recombination repair; NGS, Next-generation sequencing; *PALB2^gmut^*, Germline *PALB2* mutation; *PALB2^mut^*, Somatic *PALB2* mutation; PARP, Polyadenosine diphosphate-ribose polymerase; PD-L1, Programmed Cell Death-Ligand 1 ; MSI, Microsatellite Instability; TMB, Tumor mutation burden; PFS, Progression-free survival; OS, Overall survival; MMR, Mismatch repair; BER, Base excision repair; FA, Fanconi anemia; NER, Nucleotide excision repair; NHEJ, Non-homologous end-joining; TLS, DNA translesion synthesis; SNVs, Single nucleotide variants ; CNVs, Copy number variations.

## References

[1] Lord CJ Ashworth A. The DNA Damage Response and Cancer Therapy. Nature (2012) 481(7381):287–94. doi: 10.1038/nature10760 22258607

[2] Breast Cancer Association C Dorling L Carvalho S Allen J Gonzalez-Neira A Luccarini C. Breast Cancer Risk Genes - Association Analysis in More Than 113,000 Women. N Engl J Med (2021) 384(5):428–39. doi: 10.1056/NEJMoa1913948 PMC7611105 33471991

[3] Teo MY Seier K Ostrovnaya I Regazzi AM. Alterations in DNA Damage Response and Repair Genes as Potential Marker of Clinical Benefit From PD-1/PD-L1 Blockade in Advanced Urothelial Cancers. J Clin Oncol (2018) 36(17):1685–94. doi: 10.1200/JCO10.1200/JCO.2017 PMC6366295 29489427

[4] Neelima Vidula NKH Blouch E Rivera A Basile E Fax R Ellisen LW. Phase II Trial of a PARP Inhibitor in Somatic BRCA Mutant Metastatic Breast Cancer. 2020. ASCO (2020). doi: 10.1200/JCO.2020.38.15_suppl.TPS1113

[5] Scarbrough PM Weber RP Iversen ES Brhane Y Amos CI Kraft P. A Cross-Cancer Genetic Association Analysis of the DNA Repair and DNA Damage Signaling Pathways for Lung, Ovary, Prostate, Breast, and Colorectal Cancer. Cancer Epidemiol Biomarkers Prev (2016) 25(1):193–200. doi: 10.1158/1055-9965.EPI-15-0649 26637267 PMC4713268

[6] De’ Angelis GL Bottarelli L Azzoni C De’ Angelis N Leandro G Di Mario F. Microsatellite Instability in Colorectal Cancer. Acta BioMed (2018) 89(9-S):97–101. doi: 10.23750/abm.v89i9-S.7960 30561401 PMC6502181

[7] Hussain M Mateo J Fizazi K Saad F Shore N Sandhu S. Survival With Olaparib in Metastatic Castration-Resistant Prostate Cancer. N Engl J Med (2020) 383(24):2345–57. doi: 10.1056/NEJMoa2022485 32955174

[8] Nadine M Tung MER Ventz S Santa-Maria CA Marcom PK Nanda R. Translational Breast Cancer Research Consortium. TBCRC 048 A Phase II Study of Olaparib Monotherapy in Metastatic Breast Cancer Patients With Germline or Somatic Mutations in DNA Damage Response Pathway Genes Olaparib Expanded. ASCO (2020).

[9] Consortium TAPG. AACR Project GENIE Powering Precision Medicine Through an International Consortium. Cancer Discov (2017) 7(8):818–31. doi: 10.1158/2159-8290 PMC5611790 28572459

[10] Karachaliou N Bracht JWP Fernandez Bruno M Drozdowskyj A Gimenez Capitan A Moran T. Association of PALB2 Messenger RNA Expression With Platinum-Docetaxel Efficacy in Advanced Non-Small Cell Lung Cancer. J Thorac Oncol (2019) 14(2):304–10. doi: 10.1016/j.jtho.2018.10.168 30472259

[11] Wang Z Zhao J Wang G Zhang F Zhang Z Zhang F. Comutations in DNA Damage Response Pathways Serve as Potential Biomarkers for Immune Checkpoint Blockade. Cancer Res (2018) 78(22):6486–96. doi: 10.1158/0008-5472.CAN-18-1814 30171052

[12] Ricciuti B Recondo G Spurr LF Li YY Lamberti G Venkatraman D. Impact of DNA Damage Response and Repair (DDR) Gene Mutations on Efficacy of PD-(L)1 Immune Checkpoint Inhibition in Non-Small Cell Lung Cancer. Clin Cancer Res (2020) 26(15):4135–42. doi: 10.1158/1078-0432.CCR-19-3529 32332016

[13] Besse B. HUDSON: An Open-Label, Multi-Drug Biomarker-Directed Phase II Platform Study in Patients With NSCLC, Who Progressed on Anti-PD (L)1 Therapy, in: WCLC 2020 Virtual (2021) (Accessed January 28-31).

[14] Su D Zhang D Chen K Lu J Wu J Cao X. High Performance of Targeted Next Generation Sequencing on Variance Detection in Clinical Tumor Specimens in Comparison With Current Conventional Methods. J Exp Clin Cancer Res (2017) 36(1):121. doi: 10.1186/s13046-017-0591-4 28882180 PMC5590190

[15] Richards S Aziz N Bale S Bick D Das S Gastier-Foster J. Standards and Guidelines for the Interpretation of Sequence Variants: A Joint Consensus Recommendation of the American College of Medical Genetics and Genomics and the Association for Molecular Pathology. Genet Med (2015) 17(5):405–24. doi: 10.1038/gim.2015.30 PMC4544753 25741868

[16] Robson M Im SA Senkus E Xu B Domchek SM Masuda N. Olaparib for Metastatic Breast Cancer in Patients With a Germline BRCA Mutation. N Engl J Med (2017) 377(6):523–33. doi: 10.1056/NEJMoa1706450 28578601

[17] Moore KN Bookman M Sehouli J Miller A Anderson C Scambia G. Atezolizumab, Bevacizumab, and Chemotherapy for Newly Diagnosed Stage III or IV Ovarian Cancer: Placebo-Controlled Randomized Phase III Trial (IMagyn050/GOG 3015/ENGOT-Ov39). J Clin Oncol (2021) 39(17):1842–55. doi: 10.1200/JCO.21.00306 PMC8189598 33891472

